# Evan syndrome as initial presentation of COVID-19 infection

**DOI:** 10.1186/s43168-022-00125-x

**Published:** 2022-05-04

**Authors:** Hamdy A. Mohammadien, Lotfy H. Abudab, Azza M. Ahmad

**Affiliations:** 1 Department of Chest Diseases, Sohag Faculty of Medicine, Sohag University, Sohag, Egypt; 2 Department of Internal Medicine, Sohag Faculty of Medicine, Sohag University, Sohag, Egypt

**Keywords:** COVID-19, Autoimmune hemolytic anemia, Immune thrombocytopenia, Evans syndrome, SARS-coronavirus-2

## Abstract

**Background:**

Evans’ syndrome (ES) is a rare and chronic autoimmune disease characterized by the concomitant or sequential association of auto-immune hemolytic anemia (AIHA) with immune thrombocytopenia (ITP), and less frequently autoimmune neutropenia with a positive direct anti-human globulin test. ES represents up to 7% of AIHA and around 2% of ITP. Studies have found that coronavirus disease 2019 (COVID-19) may be associated with various hematological complications, i.e., coagulopathies; however, finding of Evans syndrome is a novel case.

**Case report:**

A 54-year-old diabetic man complaining of fever (high grade), arthralgia and myalgia, fatigue, and dark color of urine. He was admitted to isolation sector at Sohag General Hospital on day 6 because of fever with cough, dyspnea, and progressive fatigue, and at admission, he was tachypneic, tachycardiac, jaundiced, febrile (38 °C), and hypoxemic (O2 saturations on room air was 80%). Laboratory studies showed hemoglobin (Hb) 5.43 g/dL, high reticulocyte (12.5%), ↓ed platelet count (54 × 10^3^/μl), hyperbilirubinemia and elevated C-reactive protein (CRP), D-dimer, ferritin, and lactate dehydrogenase. Markers of autoimmune diseases and screening for malignant diseases were negative. HRCT chest showed bilateral small-sized peripheral ground glass opacities in both lungs, with positive reverse transcriptase-polymerase chain reaction (RT-PCR) for SARS-CoV-2 RNA in the nasopharyngeal swab. Direct Coombs test was positive for immunoglobulin (IgG) and C3d. Evans syndrome secondary to COVID-19 was diagnosed and treatment with packed red cell (PRC) transfusions, favipiravir, dexamethasone, prednisone, ceftriaxone, enoxaparin, oral hypoglycemic, and oxygen using face mask, and then Hb value increased to 10.3 g/dL and he was discharged home without any complications.

**Conclusion:**

There are few reports of patients with concurrent COVID-19 and Evans syndrome. So, SARS-CoV-2 infection should be considered in any patient presenting with new-onset ES of unclear etiology.

## Introduction

Evans syndrome (ES), which was first described in 1951, is an autoimmune disorder characterized by the development of autoimmune hemolytic anemia (AIHA) and immune thrombocytopenia (ITP) and/or immune neutropenia simultaneously or consecutively in the absence of any underlying cause [1]. The precise pathophysiology is not entirely understood; it is believed that dysregulation of the immune system is a primary contributor to the condition. It is classified as primary and secondary. Primary Evans syndrome with no cause is very rare and is seen in children. Secondary Evans syndrome may be associated with or show other diseases or conditions such as autoimmune disorders, lymphoproliferative disorders, or primary immunodeficiencies [2], viral infections including hepatitis C, cytomegalovirus, varicella-zoster, and Epstein-Barr viruses [3, 4]. ES is one of the rare presenting features of autoimmune disorders, especially systemic lupus erythematosus (SLE). Evans syndrome occurs in patients with severe multisystem SLE manifestations and sometimes may even precede the onset of disease [5]. ES is a rare condition because it is diagnosed in only 0.8 to 3.7% of all patients with either ITP or AIHA at onset. The clinical features include fatigue, pallor, jaundice, ecchymosis, petechiae, gingivorrhagia, epistaxis and mucosal bleeding, with remissions and exacerbations during the person’s lifetime, and acute manifestations as catastrophic bleeding and massive hemolysis [6]. As in other autoimmune cytopenias, there is no established evidence-based treatment and steroids are the first-line therapy, with intravenous immunoglobulin administered as a life-saving resource in cases of severe immune thrombocytopenic purpura manifestations. Second-line treatment for refractory ES includes rituximab, mofetil mycophenolate, cyclosporine, vincristine, azathioprine, sirolimus, and thrombopoietin receptor agonists. In cases unresponsive to immunosuppressive agents, hematopoietic stem cell transplantation has been successful, although it is necessary to consider its potential serious adverse effects [7, 8]. Coronavirus disease 2019 (COVID-19) is an infection caused by severe acute respiratory syndrome coronavirus 2 (SARSCOV-2). Although acute respiratory distress syndrome (ARDS), cardiac complications, and thromboembolic events have contributed to majority of the disease mortality, it has been suggested that this infection has several hematological abnormalities that develop after or concomitantly to COVID-19 infection [9] and include reduced numbers of peripheral blood lymphocytes (lymphopenia) and eosinophils with an increased polymorphonuclear-to-lymphocyte ratio, autoimmune hemolytic anemia (AIHA), immune thrombocytopenia (ITP), Evans syndrome, autoimmune neutropenia, thrombotic thrombocytopenic purpura (TTP), antiphospholipid syndrome, coagulopathies, including disseminated intravascular coagulation, and increased ferritin and d-dimer levels [10, 11].

## Case presentation

**Fig. 1:**
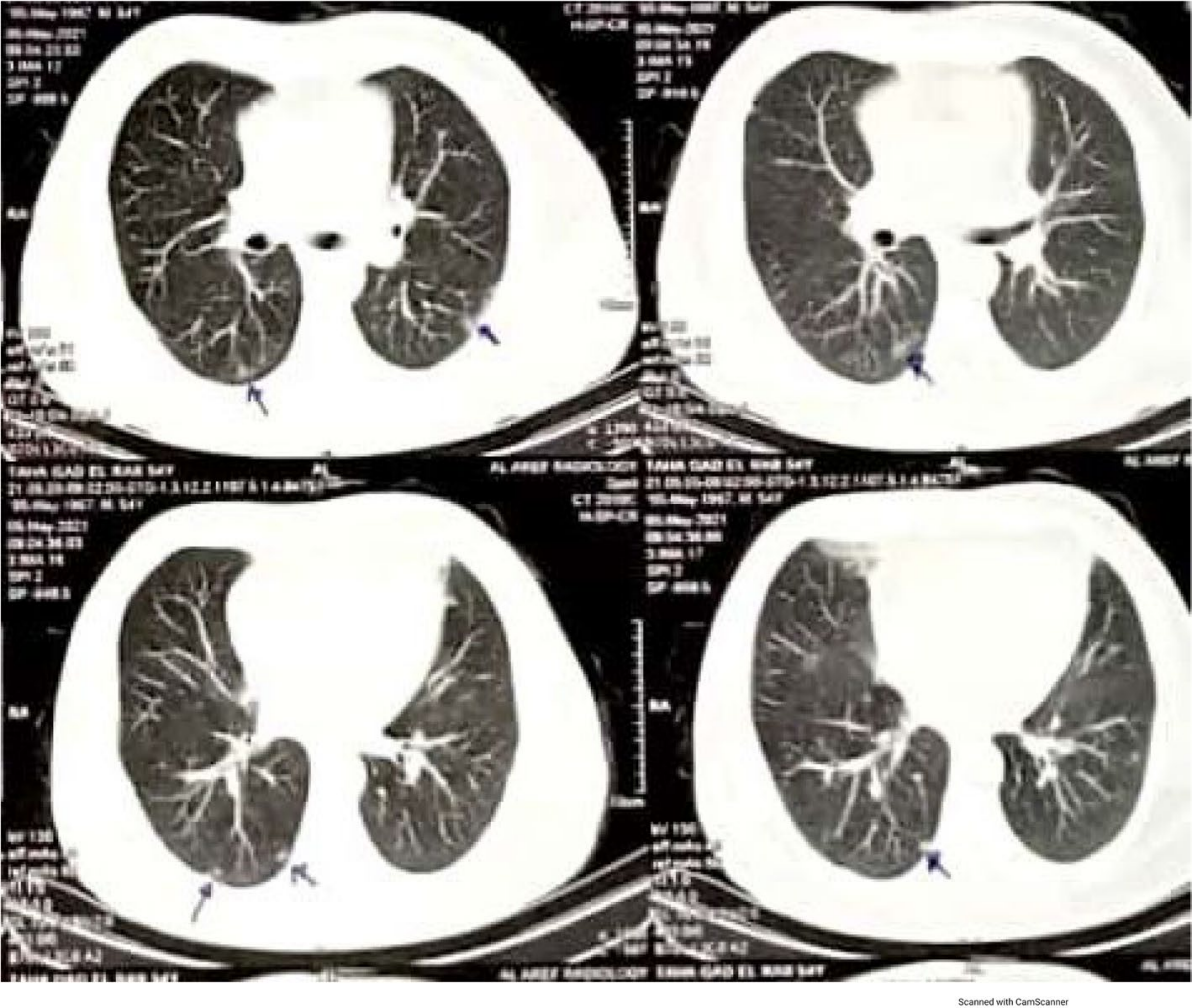
Bilateral small-sized peripheral ground glass opacities in both lungs

A 54-year-old male with a medical history of DM, Goza smoker complaining of fever (high grade), arthralgia, and myalgia, fatigue, and dark color of urine on 30 April 2021, patient sought medical advice. On physical examination, he had fever (temperature 39 °C), pallor, jaundice, no enlarged lymph node, chest, and abdominal examination was unremarkable. His oxygen saturation (SpO2) on ambient air was 98%. and his respiratory rate was 19 cycle/min, heart rate 110 beats/min. the following investigations were performed complete blood picture, Hgb 6.1, RBCs 2.23, MCV 85, platelet 185,000, WBCs 10.1, neutrophil 67%, and lymphocytes 26%. Total, direct, and indirect bilirubin (6 mg, 1.8 mg, and 4.2 mg), SGPT 25 μ/l (*N* up 41 μ/l), Widal test was negative, hepatitis markers, hepatitis A virus (HAV), IgM antibodies, hepatitis C virus antibody (anti-HCV IgG), hepatitis B virus surface antigen (HBsAg), and total core antibody were negative. Patient was given two blood transfusion and symptomatic treatment. There is no past history of blood transfusion or any bleeding problem or liver disorder in family. He was not on any medication except regularly used oral hypoglycemic drug; after 3 days, 3 May 2021, patient was reevaluated by CBC, which revealed that Hgb ↓ to 5.9 g/dl, RBCs ↓ 2.1, platelet 191,000, WBCs ↑ 13.3, neutrophil 72%, and lymphocytes ↓ 20%. Reticulocyte count 3.65% (*N* 0.5–2.0%). Total, direct, and indirect bilirubin ↑ed (7 mg, 2.4 mg, and 4.6 mg), SGPT 24 μ/l (*N* up 41 μ/l), RSG 430 mg/dl.no evidence of active bleeding was found. Possibility of hemolytic anemia was suspected. On 5 May 2021, patient was referred to be reevaluated by professor of hemotology and the following investigations were performed: complete blood count showed the following: RBCs 1.58 × 100^3^/μl, Hgb 5.4 g/dL, Htc 15.1%, total leukocyte count (WBCs) 8.3 × 10^3^/μl, neutrophil 68%, 5.64, lymphocyte relative count % 24%, absolute count 1.99, and platelet count, 117 × 10^3^/μl (thrombocytopenia), with severe normocytic normochromic anemia. Peripheral blood smear showed anisopoikilocytosis, tear drop cells rouleaux formation, reticulocytosis and normoblastemia, nucleated red blood cells, and spherocytes. Our patient had reticulocytosis of 12.5% (*N* 0.5–2.5%), The following were abnormal on laboratory tests: erythrocyte sedimentation rate (ESR): 1st hour 142 mm (*N* 3–5 mm), 2nd hour 158 mm (*N* 7–15 mm), random blood glucose (RBG) 430 mg/dl, serum creatinine 1.2 mg/dl (*N* 0.9–1.3 mg/dl), blood urea 71 mg/dl (*N* 10–50 mg/dl), estimated GFR 79 ml/min/1.73 m^2^ (*N* ˃ 90 ml/min/1.73 m^2^). There was evidence of an acute kidney injury (AKI) stage 1, C-reactive protein (CRP): 84.2 mg/L, (*N* ˂ 6.0 mg/l), D-dimer: 2920 mg/l, (*N* less than 0.5 mg/l); and serum ferritin 540.6 ng/ml (*N* 20–110 ng/ml). There was increment in bilirubin: total bilirubin 7 mg/dl (0.2–1.0 mg/dl), direct bilirubin 2.4 mg/dl (0.0–0.3 mg/dl), indirect bilirubin 4.6 mg/dl (0.3–0.8 mg/dl), alanine aminotransferase (SGPT) 24 μ/l, *N* up to 41 μ/l, and lactate dehydrogenase (LDH) of 947 U/L (35–460 μ/l). Markers of autoimmune diseases (antinuclear antibodies (ANA), anti-double-stranded DNA antibodies, ANCAc, ANCAp, and rheumatoid factor) were also negative. In addition, malignant diseases were screened and found to be negative. Chest X-ray (CXR) was within normal limits, and ultrasound of abdomen was normal. Provisional diagnosis of hemolytic anemia and thrombocytopenia with possibility of COVID-19 infection was made. On May 6 2021, (6 days later) patient develop dyspnea, cough, and progressive fatigue, he was admitted to isolation sector at Sohag General Hospital, and clinical examination revealed yellow discoloration in his eyes, no petechiae, ecchymosis, or rash. His vital signs were as follows: temperature (38 °C), pulse 112/min, respiratory rate of 25 breaths/min, and O2 saturations on room air was 80%. Chest and abdominal examination was unremarkable. Then, the patient underwent a HRCT chest which demonstrated bilateral small-sized peripheral ground glass opacities in both lungs. (Fig. 1). Reverse transcriptase-polymerase chain reaction (RT-PCR) assay detected the presence of SARS-CoV-2 RNA in the nasopharyngeal swab. Direct and indirect Coombs tests were performed as well, and the direct Coombs test was positive for immunoglobulin (IgG) and C3d, but indirect Coombs test was negative.

Combination of autoimmune hemolytic anemia (AIHA), immune thrombocytopenia (ITP), and positive direct Coombs test to IgG and C3d thus the diagnosis of Evans syndrome secondary to SARS-CoV-2 infection (COVID-19) was made. Over the course of his admission, the patient received 5 units of packed red blood cells (PRBC), antiviral treatment with favipiravir (200 mg) tab 1600 mg twice daily on day 1, followed by 600 mg twice daily for a total duration of 5 days, ivermectin (Iverzine) tab 6 mg six tab as single dose day 0, day 3, day 6, and dexamethasone 8 mg IV twice a day at the first 5 days and once for 5 days, and then prednisone 1 mg/kg daily (total daily dose of 60 mg), cefatriaxone 1 g IV once a day, moxiflox 400 mg oral once daily, enoxaparin (clexan) 60 mg SC twice daily for 10 days, receive revaroxeban tab 10 mg daily and rovitan sachets twice, oral hypoglycemic (amaryl tab 3 mg twice plus metformin (cidophage) tab 750 mg once, vitamin C 500 mg oral twice a day, along with other symptomatic medicine, and was given oxygen using face mask. Follow-up CBC on 9 May 2021 shows: RBCs 2.8 × 100^3^/μl, Hgb 9.1 g/dL, MCV 91.6, HCT 25.3%, total leukocyte count (WBCs) 13.4 × 10^3^/μl, neutrophil 70.4%, lymphocyte 21.8%, and platelet count, 99 × 10^3^/μl (thrombocytopenia). The patient’s clinical condition improved, with following clinical improvements: fever subside, SO2% 98% room air, CBC parameters, and S. bilirubin direct, and indirect. On the day of discharge, 16 May 2021, his complete blood count was as follows: RBCs 3.06, Hgb 10.3 g/dL, total leukocyte count 3.8 × 10^3^, neutrophil 68%, lymphocyte 27%, and platelet count 54 × 10^3^, Reticolocyte count: 2%, total bilirubin, conjugated bilirubin, unconjugated bilirubin, CRP, D-dimer, and LDH had decreased to (2.8, 1.5, 1.3 mg/dl, 12 mg/l, 368.4 mg/l, and 618 μ/l) respectively, SO2% 98% on room air then, he got a maintenance dose of prednisolone1mg/kg/daily oral and rivaroxaban tab 10 mg daily. One week after discharge (22 May 2021) on prednisolone, his CBC: RBCs 3.5, Hgb 10.8 g/dl, HCT (hematocrit) 30.4 l/l, RDW-CV 17.1%, platelet count 70, and WBCs 5.73, mild normocytic normochromic anemia with anisopoikilocytosis, tear drop cells, pencil-shaped cells and target cells, reticulocytosis and normoblastemia, and mild thrombocytopenia. The reticolocytic count was within normal limits (2%), total, direct, indirect bilirubin, and LDH markedly decreased to 1.63, 0.5, 1.2 mg/dl, and 490 μ/l) respectively. One week after discharge, 31 May 2021, a repeat test revealed RBCs 3.8, Hgb 11.1 g/dl, HCT 33.2 L/l, RDW-CV 15.4%, platelet count 114, and WBCs 6.7. Normocytic normochromic anemia with anisocytosis, mild thrombocytopenia, and normal reticolocytic count (1.5%) and LDH 417 μ/l. A steroid taper was planned. Follow-up laboratories 4 weeks 17 June 2021, after discharge demonstrated complete hematologic response with Hgb 12.5 g/dL, RBCs 4, HCT 35.1, MCV 87.5, WBCs 4.8, neutrophils 78%, lymphocytes 20%, platelet 67,000, normocytic normochromic anemia with (erythrocytes show) anisocytosis, thrombocytopenia with some giant platelet forms and normal reticulocyte count (0.5%) and normal bilirubin (total 1.11 mg, direct 0.34 mg, indirect 0.77 mg) and LDH 340 μ/l. serum ferritin 357.1 ng/ml. The patient was continued on a prednisone taper (Tables 1 and 2).

**Table 1:** Laboratory tests results

Indirect Coombs	Direct Coombs	Lactate dehydrogenase	Indirect bilirubin	Direct bilirubin	Total bilirubin	Ferritin	D-dimer	C-reactive protein	Lymphocytes relative % absolute	Neutrophils relative % absolute	Differential	Total count
Negative	IgG +ve C3d +ve	–	4.6 mg	2.4 mg	7 mg	–	–	–	26 2.6	67 6.7		10.06
–	–	947 U/L	–	–	–	541 ng/ml	2920	84.2	24 1.99	68 5.64		8.3
–	–	–	–	–	–	–	–	–	21.8 2.9	70.4 9.5		13.4
–	–	618 μ/l	1.3	1.5	2.8 mg	850 ng/ml	368.4	12 mg	27 1.03	68 2.6		3.8
–	–	490 μ/l	1.2	0.5–	1.63	522.1 ng/ml	–	–	28 1.6	62 3.55		5.73
–	–	417 μ/l	–	–	–	612 ng/ml	–	–	22% 1.5	76% 5.1		6.7
–	–	340 μ/l	0.77	0.34	1.11	357.1	–	–	20% 0.96	78% 3.74		4.8

Indirect Coombs	WBCs	Platelet count	Reticulocyte	Peripheral blood smear	RDW-CV	MCHC	MCH	MCV	HCT	Hgb	RBCs	CBC
Negative		185	3.65%	–	15.7	32.2	27.5	85	19.02	6.1	2.23	**3-5-21**
–		117	12.5%	Anisopoikilocytosis spherocytes	18.5	35.6	34.2	95.9	15.1	5.4	1.58	**5–5**
–		99	7.5%	–	20.3	35.9	32.9	91.6	25.3	9.1	2.76	**9–5**
–		54	2%	–		30.6	33.7	110.1	33.7	10.3	3.06	**16–5**
–		70	1.2	Anisopoikilocytosis, tear drop cells	17.1	35.5	30.9	86.9	30.4	10.8	3.5	**22–5**
–		114	1.5%	Anisocytosis	15.4	33.4	29.5	88.3	33.2	11.1	3.8	**31–5**
–		67	0.5%	Anisocytosis	13.5	35.6	31.2	87.5	35.1	12.5	4.01	**17–6**

All dates refer to the year of 2021

**Table 2:** Autoimmune hematologic complications (Evans syndrome) of SARS-CoV-2 infections

Author	Month	Country	History of the patient	Clinical presentations	Timing of the hematologic presentations	Autoimmune disorder	Treatment	Outcome
Li et al. [12]	Late March 2020	USA	39-year-old male	First admission: fever, chills, dyspnea, hemoptysis, epistaxis, sore throat, productive cough, tachycardia, tachypnea, oral blood blister, hematemesis, melena, hematochezia and no petechiae, ecchymosis or rash Second admission (10 days later, 4 days after first discharge): intermittent fever,cough, extreme weakness, fatigue, and no bleeding	About 7 days	Evans syndrome	First admission: proton pump inhibitor, IVIG Second admission: IVIG	Recovered (in first admission that patient had ITP, resolution of bleeding and raise of Plt occurred on day 5 and the patient was discharged on day 6; Hb drop also responded to IVIG in second admission)
Wahlster et al. [13]	April 2020	USA	17 -year-old male K/C of refractory chronic ITP on eltrombopag and mycophenolate mofetil	Fever, fatigue, emesis, diarrhea, progressive jaundice, marked pallor, tachycardia, tachypnea, and hypoxemia	4 days	Evans syndrome	Steroid, packed cell transfusion	Recovered (Hb became stable within 48 h of steroid administration)
Vadlamudi et al. [14]	June 2020	USA	23-year-old female gravida 2, para 1, at 38 weeks of pregnancy in active labor	Spontaneous rupture of membranes, contractions, blood-tinged discharge, history of ecchymosis and an episode of epistaxis 2 weeks prior, no pallor, ecchymosis or organomegaly On day 38 of postpartum: chest pain and shortness of breath	Not clear	Evans syndrome	IV iron dextran, IVIG, rituximab, dexamethasone, packed cell and Plttransfusion, folate (1 mg daily) and B12 (1000 mcg monthly)	Recovered
Demir et al [15]	April 26, 2020	Turkey	A 22-year-old male patient	Jaundice, weakness, shortness of breath, fever, tachycardia, tachypnea, O2 sat: 89%; and body mass index: 32.5, icteric sclerae, pale conjunctivae.	Not clear	Evans syndrome	Patient was treated with hydroxychloroquine, moxifloxacin and favipiravir for 5 days, Subcutaneous enoxaparin 1 × 0.6 cc, continuous positive airway pressure was administered intermittently, Methylprednisolone 1 mg/kg, folic acid, vitamin B12, and a proton pump inhibitor, 2 units of erythrocyte suspension daily, intravenous immunoglobulin (IVIG) 1 g/kg/day.	Recovered (on day 5 after discharge from hospital, his hemoglobin was 13 g/dL and his platelet count was 210 × 109 /L. Furthermore, his rapid antibody test (serological test) was positive for IgM and IgG against SARS-CoV 2).
Zarza et al [16]	March 23, 2020	Paraguay	A 30-year-old woman	At the time of her first visit March 23rd 2020, she presented with upper respiratory symptoms, nasal congestion, a sore throat, a cough, and the loss of her taste and smell. Medical history for a deep venous thrombosis of the right lower limb that she experienced when she was 11 years old. On April 1st, 2020, gingivorrhagia, which was self-limited. On April 5th, incoercible epistaxis appeared, Petechiae were found on her skin all over her body.	About 10 days	Evans syndrome	1 g of methylprednisolone intravenously (IV) each day for three consecutive days was started, resulting in a decrease in bleeding and purpura. Empirical treatment was started with 500 mg of azithromycin PO on day 1 followed by 250 mg per day for 4 days, 400 mg of hydroxychloroquine PO every 12 h on day 1 followed by 200 mg PO every 12 h for the next 4 days, 100 mg of prednisone PO once daily, and 1 g of ceftriaxone IV every 24 h. enoxaparin at prophylactic doses of 40 mg every 24 h. She was discharged with 50 mg of prednisone daily, 200 mg of hydroxychloroquine every 12 h, and 40 mg of enoxaparin daily. A close follow-up was indicated by all of the specialists involved in her care.	The progressive improvement of the patient’s health permitted her to be sent home.
Barcellini et al. [17]	March 25, 2020	Italy	78-year-old male	On March 25, 2020, he presented at the outpatient clinic with typical symptoms of COVID-19 pneumonia (fever, dyspnoea, desaturation to 80%). His past medical history consisted of arterial hypertension, previous myocardial infarction with ventricular fibrillation, stroke, two septic shocks, and osteonecrosis of the femoral head.	Not mentioned	Evans syndrome	Low-flow oxygen support, steroids, hydroxychloroquine (HCQ), azithromycine, full-dose LMWH, and empirical antibiotic therapy for superimposed bacterial infection.	The patient rapidly recovered from pneumonia but experienced two complications: paroxysmal atrial fibrillation treated with amiodarone, and wAIHA relapse that required IvIg and full-dose steroid (prednisone 1 mg/kg/day for 3 weeks followed by slow tapering, still ongoing).
Georgy et al. [18]	July 2020	India	A 33-year-old man	Presented to the emergency department with a 3-week history of gum bleeding, black tarry stools, and reddish spots on the skin, no fever, cough, or dyspnea, petechial lesions over the chest, legs, and oral mucosa, Within a few hours of admission, the patient complained of sudden-onset headache and developed a generalized tonic–clonic seizure. The patient’s sensorium worsened rapidly with anisocoria,	3 weeks	Evans syndrome	He was shifted to the intensive care unit, he was treated with pulse dexamethasone 40 mg daily with platelet transfusions (intravenous immunoglobulin [IVIG] was not feasible),he had not received anticoagulation	Despite the above measures, there was no improvement in the patient’s platelet counts nor sensorium, and he died on the third day of admission died
Current study	May 3, 2021	Egypt	A 54-year-old male	Fever, arthralgia, myalgia, fatigue, and dark color of urine, pallor, jaundice, and then patient develop dyspnea, cough, and progressive fatigue Yellow discoloration of eyes, tachycardia, tachypnea, O2 sat: 80%.	Hematologic manifestations were the presenting symptoms from the start	Evans syndrome	Packed red blood cells, Favipiravir (200 mg) tab, Ivermectin (6 mg) tab, Dexamethasone 8 mg IV, cefatriaxone 1 g IV, moxiflox 400 mg, enoxaparin (clexan) 60 mg SC twice, oxygen using face mask, oral hypoglycemic (amaryl tab 3 mg plus metformin tab 750, Vitamin C 500 mg, along with other symptomatic medicine.	Recovered

## Discussion

Evans syndrome (ES) is a rare condition characterized by the combination of autoimmune hemolytic anemia (AIHA) and immune thrombocytopenic purpura (ITP) [8]. Evans syndrome seems to be a disorder of immune regulation but the exact pathophysiology is unknown. Autoantibodies targeted at different antigenic determinants on red cells and platelets leading to the development of autoimmune hemolytic anemia (AIHA) and immunethrombocytopenia (ITP). Those causing red blood cell (RBC) destruction are directed against a base protein portion of the Rh blood group, while those that destroy platelets are frequently directed against platelet GPIIb/IIIa [19].

The pathogenesis and management of ES in the setting of the inflammatory milieu of COVID-19 has not been previously described and represents a unique challenge in clinical management. The exact pathophysiology of ES is not fully elucidated, but studies suggest the intersection of autoimmunity and predisposing immune dysregulation is involved. Several proposed mechanisms of autoimmunity have been described, including activation of Bruton tyrosine kinase and overexpression of cytokines [20].

Various viral infections are known to cause ES, hepatitis C virus (HCV), Epstein-Barr virus (EBV), cytomegalovirus (CMV), and Varicella Zoster Virus (VZV) [8]. More recently, SARS-CoV-2 has been reported as a potential cause of ES [11] and Li et al. [12] reported the first case of COVID-19-associated ES. This 38-year-old male patient had presented with evidence of immune hemolysis 4 days after being discharged with demonstration of a complete platelet response to treatment of ITP. Demir et al. [15] reported the second in the literature case of 22-year-old male patient presented with AIHA and grade IV thrombocytopenia, the patient was diagnosed with SARS-CoV-2-associated ES. Our patient is similar to Demir et al. [15] but differs from Li et al. [12]; the first with regard to both clinical findings and treatment method. Our patient developed AIHA first and then immune thrombocytopenia, like in case of Demir et al. [15]**,**whereas in the case of Li et al. [12], first immune thrombocytopenia and then AIHA emerged 1 week after. Another difference between the cases was that hemoglobin level in case of Demir et al. [15] was as low as 3.9 g/dL at the time of admission to the hospital despite the absence of active bleeding, so multiple erythrocyte transfusions were needed to try to increase his hemoglobin concentration. Also, Zarza et al. [16] found a case of COVID-19 associated with Evans syndrome (hemolytic anemia plus thrombocytopenia, both with autoimmune causes) and antiphospholipid antibodies.

Other autoimmune disorders associated with COVID-19 include immune thrombocytopenic purpura (ITP) and autoimmune hemolytic anemia (AIHA) [21]. COVID-19 has been identified as a causal factor of ITP in a 65-year-old woman with HTA and autoimmune hypothyroidism [22]. Other authors described the first case series of 3 patients with ITP associated with COVID-19 [23]. Lazarian et al. [10] reported seven cases of warm and cold AIHA associated with COVID-19. These cases occurred during the course of the disease earlier (i.e., a median of 9 days) after admission. However, an indolent B cell malignancy was present in four of them, all required treatment with either steroids or transfusion. Furthermore, another case of AIHA during COVID-19 was reported in a 46-year-old female with a medical history of congenital thrombocytopenia [24]. Several other hematologic disorders have been associated with COVID-19 such as cold agglutinin syndrome, Evans syndrome, or autoimmune thrombotic thrombocytopenic purpura [25, 26]. The structural similarity between an erythrocyte membrane protein named ANK-1 and the viral protein spike led Angileri et al. to postulate that molecular mimicry could contribute to the pathogenesis of COVID-19-associated AIHA [27].

Barcellini et al. [17] reported 4 patients with autoimmune cytopenias (AIC) (2 autoimmune hemolytic anemias, AIHA, 1 Evans syndrome, and 1 immune thrombocytopenia) with COVID-19 pneumonia. Most of COVID-related cytopenias described in literature developed at the time of COVID-19 infection (generally within the first month). It is largely known that autoimmune cytopenias may be triggered by viral and bacterial infections, due to mechanisms of molecular mimicry, hidden epitope spreading and neo-antigen generation [2, 28]. Interestingly, the RBC membrane protein ankyrin-1 was found to share a 100% identity with the SARS-CoV-2 surface glycoprotein spike [27], suggesting a molecular mimicry mechanism for AIHA. Regarding ITP, anti-GP IIb/IIIa, GP-Ib/IX, or GP-V antibodies have been identified in several cases [29], although a sequence homology between platelet components and SARS-CoV-2 still needs to be documented. Furthermore, during infections, platelets and viruses interact in a sialic acid-dependent manner, leading to increased hepatic clearance of platelets. Sialic acids may act as additional receptors for SARS-COV-2 spike protein, possibly accounting for thrombocytopenia in COVID-19, as observed for influenza virus [30, 31]. Finally, AIC secondary to infections are thought to be often transient and/or promptly responsive to first-line therapy.

Wahlster et al. [13] report case of a17-year-old male patient with SARS-CoV-2 infection and underlying immune dysregulation subsequently found to have ES. The onset of this patient’s COVID-19 infection was unknown; however, preexisting COVID-19 infection leading to widespread immune activation prior to her initial admission may have served as a trigger for the new onset of ES.

Vadlamudi et al. [32] reported that a 23-year-old multigravida woman in active labor was found to have severe anemia and thrombocytopenia. She was diagnosed with ES and started on immunosuppressive treatments for persistent immune thrombocytopenic purpura. In the postpartum period, she was found to have coronavirus (COVID-19) infection and acute pulmonary embolism.

Taherifard et al. [14] reported that among 94 patients with COVID-19, the most common hematologic autoimmune disorder was ITP in 55 cases (58%) followed by autoimmune hemolytic anemia (AIHA) in 22 cases (23%). Other hematologic autoimmune disorders observed in the literature include antiphospholipid syndrome (APLS) in 10 individuals, thrombotic thrombocytopenic purpura (TTP) in 3 individuals, Evans syndrome in 3 individuals, and autoimmune neutropenia in 1 individual.

Georgy et al. [18] demonstrate a 33-year-old man presented with a 3-week history of gum bleeding, black tarry stools, and reddish spots on the skin, no fever, cough, or dyspnea, with petechial lesions over the chest, legs, and oral mucosa. There was severe thrombocytopenia (6 × 109/L), anemia (7.5 g/dl), elevated lactate dehydrogenase (1953 U/L). Total and direct bilirubin were {1.23 and 0.46}, reticulocyte count was (13.73%). Direct Coombs test was positive (2+), suggesting immune hemolytic anemia. Within a few hours of admission, the patient complained of sudden-onset headache and developed a generalized tonic–clonic seizure. CT of the brain showed intracerebral hemorrhage in the right capsuloganglionic region with edema and midline shift. The patient’s sensorium worsened rapidly with anisocoria, and he was shifted to the intensive care unit. (RT-PCR) for SARS-CoV-2 was positive. There was no improvement in the patient's platelet counts, nor sensorium, and he died on the third day of admission.

Sahu et al. [33] found on their literature search 20 patients with COVID-19 who were reported to have immune dysregulation with the development of ITP, AIHA, and/or Evan’s syndrome. There were 10 (50%) patients with ITP, 9 (45%) patients with AIHA, and 1 (5%) patient had Evan’s syndrome. The average age of the patients was 61 (17–89 years) years with the majority (55%) being males (11 out of 20). Seven cases of AIHA with COVID-19 have been reported by Lazarian et al. The lowest hemoglobin (2.5 gm/dL) in AIHA with COVID-19 was reported by Wahlster et al., and Evans syndrome was reported by Li et al.

The management of Evans syndrome remains a challenge. There is no therapeutic regimen established. Steroids with and without intravenous immunoglobulin (IVIG) are recommended as first-line therapy and administered as a life-saving resource in cases of severe immune thrombocytopenic purpura manifestations. The American Society of Hematology guideline [34] recommends dexamethasone (40 mg/day for 4 days) or prednisolone (1 mg/kg/day) with tapering (depending on response and for a maximum duration of 6 weeks, red blood cell/platelet transfusion is indicated only in severe symptomatic patients. Second-line treatment for refractory ES includes rituximab, cyclophosphamide, mycophenolate mofetil, cyclosporine, vincristine, azathioprine, sirolimus, splenectomy, and thrombopoietin receptor agonists. In cases unresponsive to immunosuppressive agents, hematopoietic stem cell transplantation has been successful. Anticoagulation thromboprophylaxis with low molecular weight heparin is recommended for in-patients with acute exacerbation. This will be stopped if platelet count˂ 50 G/L [32].

The treatment of AIHA or ES during an infection is difficult and may be best individualized according to the patient’s characteristics. By sharing our experiences, as the data in the literature increase, it may become easier to make the most beneficial treatment decisions for the patient [15].

To the best of our knowledge, this is the first case report of COVID-19 infection with Evans syndrome in Egypt. In another case report, seven patients from hospitals in the USA, Italy, Turkey, India, and Paraguay reported the emergence of ES during COVID-19 infection.

## Conclusion

Our case highlights the fact that SARS-CoV-2, the causative agent of COVID-19, may itself be capable of inducing Evans syndrome even in patients with no underlying predisposition. So, SARS-CoV-2 infection should be considered in any patient presenting with new-onset ES of unclear etiology.

## Data Availability

All data and material of the case are available.
